# Comparison of Toe Clearance Characteristics Between Simulated Obstacle Crossing Using Visual Height Cues and Actual Obstacle Crossing

**DOI:** 10.3390/brainsci16020248

**Published:** 2026-02-23

**Authors:** Mao Kasai, Yumi Machida, Miku Washizu, Kenichi Sugawara, Tomotaka Suzuki

**Affiliations:** School of Rehabilitation, Kanagawa University of Human Services, Yokosuka 238-8522, Japan

**Keywords:** obstacle crossing, simulated task, toe clearance, visual cues, motor control

## Abstract

**Highlights:**

**What are the main findings?**

- Simulated obstacle crossing using visual cues successfully evoked height-dependent limb scaling but resulted in significantly reduced safety margins compared to actual crossing.
- The absence of physical risk led to a systematic underestimation of TC_min_ and increased motor variability, as noted in the quartile coefficient of variation, with the most pronounced deficits observed in the trail limb.

**What are the implications of the main findings?**

- Simple repetition in risk-free simulated environments may be insufficient for acquiring the precise motor control strategies required for safe real-world navigation.
- Effective fall prevention training should incorporate task-relevant feedback mechanisms to bridge the dissociation between motor intention and execution precision.

**Abstract:**

**Background/Objectives**: Tripping is a major cause of falls and necessitates accessible training. This study aimed to fundamentally evaluate the biomechanical fidelity of a simplified simulated obstacle-crossing paradigm using visual height cues. **Methods**: Two experiments that included healthy young adults evaluated toe clearance (TC) responsiveness during simulated crossing to four visual cue heights (Experiment 1: n = 16) and compared it with actual crossing (4–16% leg length) to assess biomechanical fidelity (Experiment 2: n = 18). Linear mixed models were used to analyze the effects of obstacle height, task condition, and walking course on vertical TC metrics, including minimum and maximum clearance and quartile coefficient of variation (QCV) for both the lead and trail limbs. **Results**: In Experiment 1, TC parameters scaled systematically with cue height (*p* < 0.001), confirming that visual cues elicited adaptive gait adjustments. In Experiment 2, although the maximum TC scaled similarly across conditions, the minimum TC was systematically reduced in the simulated condition compared to actual obstacle crossing (*p* < 0.001). Furthermore, the simulated condition exhibited increased QCV (*p* < 0.001), particularly for the trail limb at the highest obstacle height. **Conclusions**: Motor intention and execution precision were dissociated in the simulated obstacle crossing. Without physical risk, the central nervous system appeared to prioritize effort economy over the precise fine-tuning of safety margins. These results suggest that task repetition in risk-free simulations alone may be insufficient for acquiring safe obstacle-crossing strategies and highlight the importance of task-relevant feedback for ensuring biomechanical fidelity in fall-prevention research.

## 1. Introduction

Falls pose a critical threat to the health and independence of older adults, with tripping identified as a leading cause of injury during walking on both level and uneven surfaces [1,2]. This vulnerability is commonly attributed to an age-related decline in the integration of sensorimotor and executive functions, including motor planning and attentional allocation [3]. Consequently, there is growing emphasis on enhancing adaptive gait capacity, which is the ability to appropriately modify motor patterns in response to environmental demands [4].

Within this framework, obstacle crossing is a fundamental yet highly complex locomotor task that requires precise voluntary gait adjustments to ensure safe navigation [4]. The crossing process entails sophisticated sensorimotor coordination, encompassing visual feedforward planning for the lead limb and memory-guided control of the trail limb [5]. However, this feedforward control relies on executive resources. Recent evidence suggests that increased cognitive load or sensory variability competes for limited attentional resources, thereby compromising postural stability and reducing the precision of motor control [6]. Given these multidimensional demands, obstacle crossing may reflect the overall quality of motor control, and consequently, serve as an efficient and comprehensive target for fall prevention training.

Systematic reviews have shown that virtual reality-based training can improve balance and mobility and is associated with reduced fall risk in older adults [7]. To capitalize on the rehabilitative potential of obstacle crossing, studies have increasingly employed simulated training environments, particularly those that integrate virtual reality with treadmill walking [8,9,10]. Although such interventions have been shown to systematically alter gait behavior [11], whether these modifications represent optimal adaptations for safe locomotion remains unclear. Specifically, the extent to which simulated environments can elicit fine-grained sensorimotor adjustments necessary to maintain adequate safety margins remains unanswered [8]. Furthermore, laboratory-based minimum toe clearance (MTC) assessments may lack ecological validity in predicting community-based trips, because the capacity to adapt MTC to environmental challenges appears more relevant than steady-state gait characteristics alone [12]. This underscores the need to evaluate obstacle crossing not only during straight-line walking but also along more complex trajectories, such as turning, which impose greater demands on anticipatory control and sensorimotor integration than straight walking alone. A recent study has emphasized that navigating complex trajectories, such as curved or non-linear paths, places increased demands on anticipatory control, attention, and sensorimotor integration, thereby revealing aspects of adaptive gait capacity that may remain obscured during simple walking tasks [13]. Accordingly, the present study was designed as a fundamental investigation of healthy young adults to characterize adaptive gait control under reduced physical constraints. In addition to these scientific considerations, practical constraints, including excessive equipment costs and substantial space requirements, limit the accessibility of advanced simulation systems. Accordingly, a safe and low-cost training paradigm that preserves biomechanical relevance while remaining feasible for routine clinical implementation is needed.

To address these challenges, we investigated a simplified simulated obstacle crossing paradigm that utilized visual height cues placed adjacent to the walking path, rather than physical obstacles at the crossing location. In this paradigm, the obstacle height was specified using three-dimensional objects, whereas the crossing location itself was marked on the floor. This approach is easy to implement, allows immediate adjustment of obstacle parameters, and ensures safety by eliminating collision risks. Importantly, the design intentionally dissociates height specification from physical contact, reflecting a simplified but visually explicit form of obstacle representation that is conceptually consistent with prior virtual reality-based gait training research. By arranging these cues within continuous walking circuits that incorporate both straight and curved segments, the paradigm enables continuous repetitive practice, thereby securing the training volume required for motor learning. Accordingly, this paradigm was first examined in healthy young adults to characterize the fundamental motor control responses elicited by simulated obstacle crossing. To assess the validity of this approach, we focused on toe clearance (TC) because its variability is a well-established indicator of tripping risk during level walking [14].

The present study comprised two experiments involving healthy young adults, designed to comprehensively evaluate this novel paradigm. Experiment 1 examined the responsiveness of TC parameters during simulated obstacle crossing relative to four levels of visual height cues across both straight and curved paths. This study aimed to determine whether gait adjustments are systematically modulated by visual information. Experiment 2 assessed the biomechanical fidelity of the simulated task by directly comparing it with actual obstacle crossing, with particular emphasis on safety margins and movement stability. Collectively, through these experiments, we aimed to provide fundamental evidence supporting a safe, accessible, and biomechanically meaningful training strategy for fall prevention.

## 2. Materials and Methods

### 2.1. Participants

The participants were recruited via advertisements at the university. The inclusion criteria were (1) healthy young adults and (2) normal or corrected-to-normal vision. Based on these criteria, 16 university students (9 males and 7 females; age: 20–21 years) participated in Experiment 1. Experiment 2 involved 18 university students (11 males, 7 females; age: 20–22 years; mean leg length: 78.0 ± 3.8 cm). In both experiments, the participants had no history of visual, neurological, or orthopedic disorders that could affect gait or obstacle crossing, and none reported difficulty in performing the tasks. This study was approved by the Research Ethics Committee of Kanagawa University of Human Services (No. 24-34-013, 6 February 2025). Prior to the experiment, the purpose, content, and risks of the study were explained to all participants, and written informed consent was obtained.

Prior to data collection for Experiments 1 and 2, pilot studies were conducted with five healthy adults to estimate the required sample sizes using G*Power 3.1 [15]. Because the experimental protocols, environmental settings, and instructions were identical between the pilot and main experiments, and no modifications were made to the study design, the pilot data were included in the final analyses to maximize statistical power without introducing methodological bias. For Experiment 1, although the calculation indicated a required sample size of only three owing to a large effect size, we recruited 16 participants to ensure result stability and provide a sufficient safety margin. In Experiment 2, the sample size was calculated to detect differences in response patterns between the simulated and actual conditions (interaction). Based on the pilot data (f = 0.09, r = 0.87), we set the effect size (f) at 0.10, with α = 0.05 and 1-β = 0.80. The calculation yielded the required sample size of 17; consequently, 18 participants were included in the analysis.

### 2.2. Experimental Environment and Tasks

**Figure 1:**
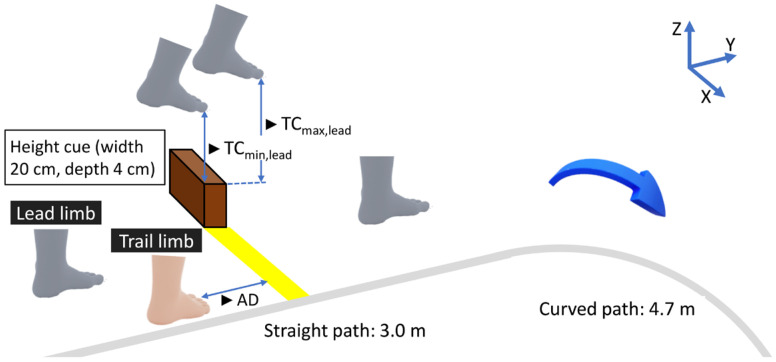
Schematic representation of the simulated obstacle-crossing task and kinematic parameters in Experiment 2. The diagram illustrates the spatial relationship between the foot, target tape, and visual height cue during a representative crossing trial. The laboratory coordinate system was defined as follows: X, mediolateral; Y, direction of progression; Z, vertical. TC: toe clearance; AD: approach distance.

The measurements were performed in a customized behavior analysis room. An oval walkway with a circumference of 15.42 m was used in this study. Four height cues (objects indicating obstacle height) or actual obstacles of different heights were placed at the centers of two straight and two curved sections (Figure 1). Considering the potential application of this task in gait training, a continuous-circuit course was used to verify the data for both the straight and curved paths.

Experiment 1 targeted only simulated obstacle crossings. Wooden blocks (width: 20 cm; depth: 4 cm) were used as cues at four heights (3, 6, 9, and 12 cm). The heights were selected to provide graded variations in obstacle height that could be negotiated during continuous walking, while allowing repeated practice under safe conditions. The height cues were placed outside the course. To mark the crossing position, yellow tape (width: 40 cm, depth: 5 cm) was affixed to the floor within the course. The participants were instructed to cross over the tape as if a physical obstacle of the same height as the height cue existed, thereby simulating an actual obstacle-crossing maneuver.

Experiment 2 included two task conditions: simulated and actual crossing. To standardize the difficulty across participants, obstacle heights were set relative to each participant’s leg length. Leg length was defined as the trochanteric height measured in the supine position prior to the experiment. The heights were set at 4%, 8%, 12%, and 16% of the leg length to approximately correspond to the fixed values used in Experiment 1. The height of the wooden obstacles was adjusted in 0.5 cm increments. The size and placement of the height cues in the simulated conditions were the same as those used in Experiment 1. Under actual conditions, obstacles (width: 40 cm, depth: 4 cm) were placed on the course.

### 2.3. Experimental Procedure

The participants walked barefoot around the course in a clockwise direction at a self-selected speed. They were instructed not to predetermine a lead limb but to repeat the crossing motion as naturally as possible according to the obstacle height. The participants crossed four obstacles per lap, with each set consisting of five continuous laps. Rest intervals were provided between the sets. The arrangement of the four obstacle heights for each set was determined using a computer-generated pseudo-random sequence to ensure that each height appeared at every location with equal frequency.

Experiment 1 consisted of eight sets of simulated conditions for 160 crossing trials. Experiment 2 consisted of simulated and actual conditions performed in two blocks of four sets each, alternating between conditions for 16 sets and 320 crossing trials. The order of the conditions was counterbalanced across participants (i.e., half of the participants started with the simulated condition, and the other half started with the actual condition). Experiment 2 took approximately 60 min to complete.

### 2.4. Data Collection and Processing

Obstacle-crossing movements were recorded at a sampling frequency of 200 Hz using a 10-camera motion capture system (Vicon MX; Vicon Motion Systems Ltd., Oxford, UK). Reflective markers were attached bilaterally to the dorsal surface of the distal phalanx of the hallux. In Experiment 1, the height cues were positioned parallel to the target tape such that their anterior edges were aligned. Reflective markers were placed on the floor at both lateral ends of the combined anterior edge line. In Experiment 2, the markers were attached to the lateral aspects of the top and anterior edges of the height, or to actual obstacles. Marker trajectories were recorded and processed using Nexus v2.10.3 (Vicon Motion Systems Ltd.) and smoothed using a Woltring filter (generalized cross-validatory spline smoothing) with a mean squared error parameter of 10 mm^2^, which is the standard setting in the Vicon Nexus pipeline and has been widely used in gait analysis studies [16]. Prior to each data collection session, the motion capture system was calibrated using a dynamic wand, ensuring that average residual errors were less than 1.0 mm. The output data were converted to the CSV format and processed offline using a custom calculation sheet in Microsoft Excel.

TC, the primary outcome measure, was calculated as follows. First, the horizontal distance between the toe marker and the anterior edge line was calculated using the point-to-line distance formula based on the XY coordinates of the toe marker and the two markers defining the edge line. The frame with the minimum horizontal distance was defined as the crossing time.

In Experiment 1, the vertical distance of the toe marker from the floor was calculated as the MTC. In addition, the maximum vertical distance of the toe marker around this time point was calculated as the peakTC. In Experiment 2, the vertical clearance between the toe marker and the upper edge of the obstacle (or height cue) was calculated at the crossing time (TC_min_) and maximum toe elevation (TC_max_). To ensure data reliability, all detected timings were plotted on kinematic waveforms and visually inspected to identify outliers owing to detection errors. No trials were excluded from the analysis because of marker-tracking errors. Furthermore, to clarify the variability of these parameters, the quartile coefficient of variation (QCV = (Q3 − Q1)/(Q3 + Q1)) was calculated. Additionally, the horizontal distance between the toe marker of the trail limb and the anterior edge line immediately before the obstacle was calculated as the approach distance (AD).

### 2.5. Statistical Analysis

All TC parameters were calculated and analyzed separately for the lead and trail limbs. In Experiment 1, the dependent variables were MTC_lead_, MTC_trail_, peakTC_lead_, and peakTC_trail_. In Experiment 2, the dependent variables were TC_min,lead_, TC_min,trail_, TC_max,lead_, TC_max,trail_, and QCVs. The QCV data were log-transformed prior to analysis to correct for positive skewness and to meet the assumptions of the linear mixed model (LMM). AD was analyzed as a secondary outcome, and the results are presented in Supplementary Figure S1.

These parameters were applied to the LMMs in all the obtained trials. In Experiment 1, the fixed effects were obstacle height (3, 6, 9, and 12 cm), walking course (straight vs. curved), and their interaction. In Experiment 2, the fixed effects were task condition (actual vs. simulated), obstacle height (4%, 8%, 12%, and 16% of leg length), walking course (straight vs. curved), and their interactions. Participants were included as random effects, and walking time and leg length (Experiment 2 only) were entered into the model as covariates. The models were estimated using Restricted Maximum Likelihood. Residual normality and homogeneity were visually confirmed in all models. Fixed effects were assessed using Type III analysis of variance with Satterthwaite’s method for degree of freedom approximations. If the interactions were significant, simple main-effect analyses were performed. If a significant effect of height was observed, Bonferroni-corrected multiple comparisons were performed for adjacent pairs.

Statistical significance was set at *p* = 0.05. All statistical analyses were performed using the lme4, lmerTest, emmeans, and effect size packages in R (version 4.5.2) and RStudio (version 2025.09.2+418). Effect sizes were estimated using partial eta squared (η_p_^2^) and interpreted according to Cohen’s guidelines (0.01: small, 0.06: medium, 0.14: large) [17].

## 3. Results

Detailed results of the LMM analyses, including effect sizes (η_p_^2^) for all tested effects, are presented in Supplementary Tables S1–S3. Visual inspection of the residual plots confirmed that the assumptions of normality and homoscedasticity were satisfied for all the models. Regarding the covariates, walking time showed a significant main effect on toe clearance parameters (*p* < 0.001), with shorter walking times consistently associated with reduced clearance values. This result justifies the inclusion of walking time as a covariate to statistically control for variations in gait speed.

### 3.1. Experiment 1

#### 3.1.1. Lead Limb

**Figure 2:**
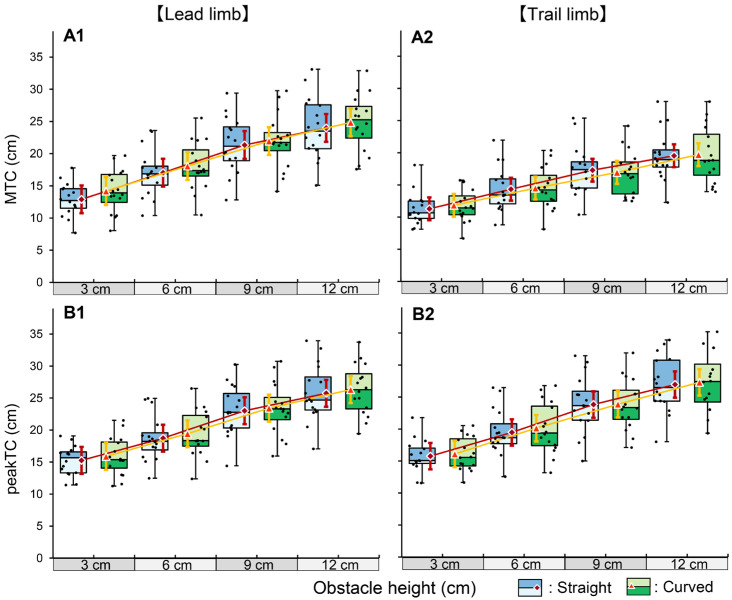
Effect of obstacle height and walking course on toe clearance (TC) parameters in Experiment 1. The box plots with jittered points represent the distribution of raw data for the straight (blue) and curved (green) paths. The red diamonds and orange triangles indicate the estimated marginal means for the straight and curved paths, respectively. Error bars represent 95% confidence intervals. The panels show the results for minimum TC (**A1**,**A2**) and peakTC (**B1**,**B2**) of the lead limb (left column) and trail limb (right column). For all parameters, a significant main effect of obstacle height was observed, and post hoc comparisons confirmed that TC significantly increased with each increment in obstacle height (all adjacent pairs: *p* < 0.001).

For both MTC_lead_ and peakTC_lead_, no significant interaction was observed between obstacle height and walking course (Figure 2). Consequently, the main effects were analyzed. A significant main effect of obstacle height was observed for both parameters (*p* < 0.001). Post hoc comparisons with Bonferroni correction revealed that toe clearance significantly increased with each increment in obstacle height (all adjacent pairs: *p* < 0.001). A significant main effect of walking course was also observed (*p* < 0.001); specifically, the MTC_lead_ was significantly increased on the curved path compared to the straight path (mean difference: 0.94 cm).

#### 3.1.2. Trail Limb

Similar to the lead limb, no significant interaction was observed for MTC_trail_ or peakTC_trail_ (Figure 2). A significant main effect of obstacle height was confirmed with stepwise increases across all heights (*p* < 0.001). Regarding the walking course effect, although statistical significance was found for peakTC_trail_ (*p* < 0.001), the effect size was negligible (η_p_^2^ = 0.002; Table S1), and no significant main effect was observed for MTC_trail_ (*p* = 0.077).

### 3.2. Experiment 2

#### 3.2.1. Lead Limb

**Figure 3:**
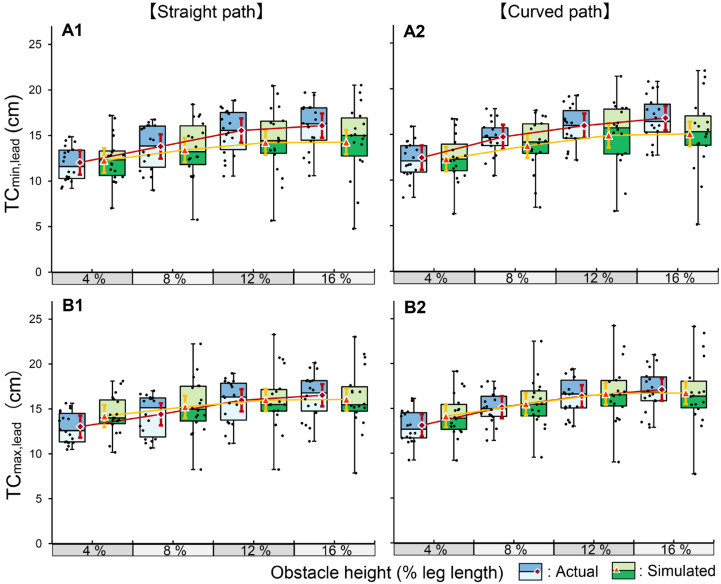
Comparison of actual and simulated obstacle-crossing kinematics for the lead limb in Experiment 2. The box plots with jittered points represent the distribution of raw data for the actual (blue) and simulated (green) crossing conditions across four relative obstacle heights (4%, 8%, 12%, and 16% of leg length). The red diamonds and orange triangles indicate the estimated marginal means for the actual and simulated conditions, respectively. Error bars represent 95% confidence intervals. The panels show the results for TC_min,lead_ (**A1**: straight path, **A2**: curved path) and TC_max,lead_ (**B1**: straight path, **B2**: curved path). TC: toe clearance.

For TC_min,lead_, the LMM analysis revealed no significant three-way interaction among condition, height, and course (*p* = 0.119; Figure 3). However, a significant two-way interaction was found between condition and obstacle height (*p* < 0.001, η_p_^2^ = 0.015). A simple main effect analysis indicated distinct response patterns between the two conditions. Although TC_min,lead_ significantly increased with obstacle height in the actual condition across all levels (*p* < 0.001), the increase between 12% and 16% height was not significant in the simulated condition (*p* = 1.000), suggesting a saturation effect in the simulated task. Furthermore, comparing the two conditions at each height, TC_min,lead_ was significantly reduced in the simulated condition compared to the actual condition at all heights, except 4% (*p* < 0.001), indicating a systematic underestimation of the required clearance. Regarding the walking course effect, although statistical significance was observed (*p* < 0.001), the effect size was small (η_p_^2^ = 0.009), with values on the curved path being slightly increased (mean difference: 0.5 cm) compared to the straight path.

For TC_max,lead_, the three-way interaction was not significant (*p* = 0.284), whereas the interaction between condition and obstacle height was significant (*p* < 0.001, η_p_^2^ = 0.010). Consistent with TC_min,lead_, the simulated conditions exhibited significantly reduced values compared to the actual conditions at higher obstacle heights. Although a significant interaction between height and course was detected, the effect size was negligible (*p* = 0.010, η_p_^2^ = 0.002; Table S2).

#### 3.2.2. Trail Limb

**Figure 4:**
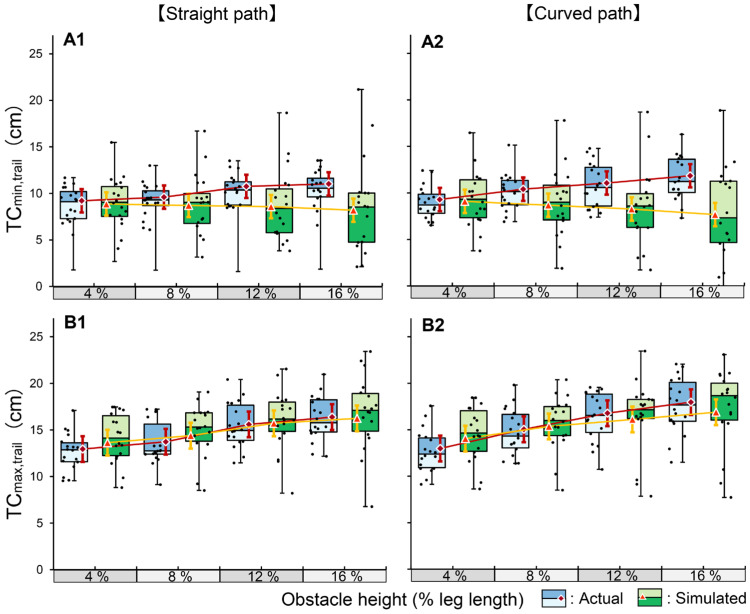
Comparison of actual and simulated obstacle-crossing kinematics for the trail limb in Experiment 2. The box plots with jittered points represent the distribution of raw data for the actual (blue) and simulated (green) crossing conditions across four relative obstacle heights (4%, 8%, 12%, and 16% of leg length). The red diamonds and orange triangles indicate the estimated marginal means for the actual and simulated conditions, respectively. Error bars represent 95% confidence intervals. The panels show the results for TC_min,trail_ (**A1**: straight path, **A2**: curved path) and TC_max,trail_ (**B1**: straight path, **B2**: curved path). TC: toe clearance.

For TC_min,trail_, the three-way interaction was not significant (*p* = 0.064; Figure 4). However, a significant interaction was found between condition and obstacle height (*p* < 0.001, η_p_^2^ = 0.025). Simple main effects analysis revealed contrasting strategies; in the actual condition, clearance significantly increased with every increment in obstacle height (4% vs. 8% and 8% vs. 12%, *p* < 0.001; 12% vs. 16%, *p* = 0.026). In contrast, in the simulated condition, clearance significantly decreased at the 16% height compared to the 12% height (*p* = 0.040). Furthermore, TC_min,trail_ was significantly reduced in the simulated condition compared to the actual condition at all heights except 4%, and this discrepancy widened as obstacle height increased (all *p* < 0.001). A significant interaction between condition and course was also observed (*p* < 0.001), but the effect size was negligible (η_p_^2^ = 0.002).

For TC_max,trail_, the three-way interaction was not significant (*p* = 0.154). Although significant two-way interactions were detected for condition × height (*p* < 0.001, η_p_^2^ = 0.007) and height × course (*p* = 0.020, η_p_^2^ = 0.002), their effect sizes were extremely small. Consequently, the main effects were dominant. A significant main effect of obstacle height was observed (*p* < 0.001), with stepwise increases across all levels. A significant main effect of course was also found (*p* < 0.001, η_p_^2^ = 0.009); values on the curved path were slightly increased compared to the straight path (mean difference: 0.7 cm), although the effect size was small.

#### 3.2.3. Variability of TC

The QCV results are presented in Table 1. For TC_min,lead_ and TC_max,lead_, no significant interactions were observed. However, a significant main effect of task condition was found (*p* < 0.001), with the simulated condition demonstrating significantly increased variability compared to the actual condition.

For TC_min,trail_, a significant interaction between condition and obstacle height was observed (*p* < 0.001, η_p_^2^ = 0.063). Simple main effects analysis indicated that, in the simulated condition, variability significantly increased at the 16% height compared to the 12% height (*p* < 0.001), whereas no such increase was found in other pairs. Additionally, the simulated condition exhibited significantly increased variability compared to the actual condition at all heights (8%, *p* = 0.002; others, *p* < 0.001). For TC_max,trail_, no significant interactions were found. A significant main effect of task condition was observed (*p* < 0.001, η_p_^2^ = 0.137), with the simulated condition showing increased variability. A significant main effect of height was also found (*p* = 0.037, η_p_^2^ = 0.033), where variability increased at 8% compared to 4% (*p* = 0.011).

## 4. Discussion

Although simulated obstacle crossing offers a safe and accessible approach relevant to fall-prevention research, its biomechanical fidelity has not been established, particularly regarding whether simplified visual cues can elicit gait adaptations comparable to those observed during actual obstacle crossing. To address this gap, the present study was designed as a fundamental evaluation of a simplified simulated obstacle-crossing paradigm, focusing on adaptive gait control under reduced physical constraints.

### 4.1. Experiment 1: Basic Kinematic Patterns in Simulated Obstacle Crossing

The primary aim of Experiment 1 was to verify whether fundamental kinematic response patterns could be elicited in a simulated obstacle-crossing task using height cues. The results demonstrated that participants successfully modulated their limb trajectories in response to the cue height. Specifically, as the cue height increased, the linear increase observed in MTC_lead_ was consistent with previous findings from studies involving obstacle crossing [18,19].

MTC_trail_ also increased monotonically, although the rate of increase was less pronounced than that of the lead limb. This blunted scaling suggests that the trail limb is less sensitive to variations in obstacle height. Chou and Draganich [20] reported that the trail-limb clearance remained unchanged with increasing obstacle height, whereas Kunimune et al. [19] reported that the clearance scale was less robust than that of the lead limb. The pattern observed in the present study aligned with those findings.

Across all cue heights, MTC was consistently reduced in the trail limb compared to the lead limb. This discrepancy is a well-documented characteristic of obstacle negotiation that is attributed to the lack of online visual feedback for the trail limb, and the present results are consistent with those of previous studies [18,19]. Furthermore, the stepwise increase in peakTC for both limbs paralleled the kinematic changes reported in a previous study [18].

Regarding the effect of the walking path, adaptive strategies differed between the limbs. For the lead limb, the MTC was significantly increased on the curved path compared to the straight path, with no significant interaction between obstacle height and course. To the best of our knowledge, kinematic adjustments of toe clearance during obstacle crossing on curved paths have not been comprehensively documented in the literature. However, curved walking is known to impose increased motor demands and instability compared to straight walking, often necessitating reduced gait speed [21]. Therefore, the observed increase in toe clearance in the lead limb likely reflects a compensatory strategy to secure a larger safety margin during this challenging task. In contrast, no such adaptation was observed in the trail limb. This lack of adjustment suggests that the trail limb may not be finely tuned to the environmental complexity in the simulated conditions.

### 4.2. Experiment 2: Comparison Between Simulated and Actual Obstacle Crossing

#### 4.2.1. Lead Limb

For the lead limb, distinct control strategies emerged between the two conditions. In the actual condition, both TC_min,lead_ and TC_max,lead_ increased linearly with the obstacle height. This linear increase suggests that, under actual obstacle-crossing conditions, participants likely adopted a conservative safety-first strategy by expanding their safety margins in proportion to the increased risk associated with higher obstacles [5,22]. This adaptation is likely driven by the complexity of navigating multiple successive obstacles of four varying heights, which requires greater anticipatory motor planning and integration of visual information than single-obstacle negotiation [23]. Under these challenging conditions, the presence of physical obstacles has been shown to elicit a robust adaptive response that prioritizes a larger safety factor in anticipation of potential contact [5,24].

In the simulated condition, TC_max,lead_ exhibited a linear increase, similar to that observed in the actual condition, suggesting that the participants maintained the capacity and intention to elevate their limbs. However, TC_min,lead_ was systematically reduced and exhibited a saturation effect at higher obstacle levels (12–16%). The maintenance of TC_max,lead_ along with the saturation of TC_min,lead_ implies a fundamental modification of the trajectory shape. Furthermore, the simulated condition was characterized by a significantly increased QCV compared to the actual condition, reflecting the reduced movement stability when the physical constraints were removed. In the absence of a physical penalty, the central nervous system likely prioritizes energy economy over safety [25]. As the perceived risk and physical consequences of contact are removed, robust safety margins typically triggered by high-risk obstacles may not be elicited [26]. Consequently, the regulatory mechanism responsible for the precise fine-tuning of the trajectory at the anterior edge of the obstacle was abandoned, resulting in a ballistic trajectory that achieved the geometric peak height but failed to secure the critical safety clearance. Notably, these condition-specific patterns and the associated instability were consistently observed, regardless of the walking course, as evidenced by the negligible effect sizes associated with this factor on the clearance parameters.

#### 4.2.2. Trail Limb

With respect to the trail limb, a notable reversal in the control strategy was observed between the two conditions. Similar to the lead limb, TC_min,trail_ in the actual condition increased linearly with the obstacle height, reflecting a conservative safety strategy. This linear expansion is particularly striking when compared with previous reports of constant trail clearance [20] or clearance decrease at the highest obstacle heights [18]. However, this safety assurance collapsed entirely under the simulated conditions. TC_min,trail_ was significantly reduced compared to the actual condition at obstacle levels of 8% and above and decreased further as the obstacle height increased from 12% to 16%.

This collapse underscores the inherent vulnerability of trail-limb control. Unlike the lead limb, the trail limb lacks online visual feedback during the crossing phase, necessitating reliance on feedforward control based on spatial memory [27]. This blind control mode renders the trail limb fundamentally susceptible to errors, with the trail limb reportedly accounting for approximately 92% of spontaneous obstacle contacts in healthy young adults [28]. This vulnerability is further evidenced by our QCV results; although the lead limb remained relatively stable, the trail limb showed a progressive increase in variability in the simulated condition, reaching its greatest magnitude at the 16% height. Such a breakdown in safety control closely resembles the failure observed in adults in their 70s under dual-task conditions, in which a significant decrease in safety margins is selectively observed in the trail limb as attentional resources are depleted [29].

Furthermore, the preservation of TC_max,trail_ across conditions suggests a conflict between the motor intention and trajectory execution precision in the simulated environments. While the participants maintained the intention to elevate their limbs according to the target height, the absence of physical risk likely eliminated the primary functional constraint that motivated precise spatial negotiation [27]. In addition to this lack of risk, the absence of physical feedback, specifically tactile or haptic information that signals obstacle contact, prevented the recalibration necessary for accurate trail-limb trajectory control. Consequently, the removal of these constraints leaves blind control of the trail limb in a state of functional instability, in which motor control appears to favor the basic act of limb elevation over the cognitively demanding fine-tuning of the clearance trajectory. The consistent observation of these control deficits, regardless of course shape, further suggests that the identified instability is an intrinsic limitation of the simulated environment rather than a consequence of geometric task complexity.

### 4.3. General Discussion

The collective results of Experiments 1 and 2 demonstrate a clear dissociation between motor intention and execution precision in simulated obstacle crossings. Although the simulated environment successfully elicited gross kinematic patterns such as height-dependent scaling of TC_max_ and stable AD (Figure S1), it failed to replicate the fine-grained control required to secure adequate safety margins. Individuals can improve their gross gait characteristics, including crossing velocity and stride length [8], or modulate peak limb elevation and foot placement [9]. Nevertheless, as observed in the present simulated condition, such adaptations are often accompanied by a systematic underestimation of TC_min_ and pronounced variability in motor output (QCV), which is consistent with reports that simulated training frequently fails to enhance the vertical TC [8]. This dissociation likely reflects a fundamental disruption in perception–action coupling within simulated environments, wherein the linkage between perceived obstacle height and motor precision is weakened when physical risk is absent [10]. Comparable vulnerability of execution-level precision has been reported in other postural control contexts in which task-relevant sensory or functional constraints are altered, indicating that fine-grained feedforward control is particularly sensitive to reductions in available constraint information [30]. Without the functional constraints imposed by real physical consequences, the central nervous system may prioritize effort economy over the cognitively demanding fine-tuning of TC [10]. Moreover, task-specific characteristics of the simulated paradigm, including altered visual–motor relationships, may also have contributed to the observed changes in obstacle-crossing performance. Collectively, these findings suggest that, although visual cues can evoke the general geometric form of negotiation, the fidelity of the underlying control mechanism is substantially degraded when physical constraints are removed.

The present investigation was observational in nature and did not evaluate long-term training effects, nor were obstacle distances varied to prevent potential repetitive lead-limb usage. Nevertheless, the systematic underestimation of TC_min_ observed in this study is likely to persist over time in the absence of specific error-correction mechanisms. To address this precision gap and restore biomechanical fidelity, simulated environments may need to incorporate mechanisms that effectively substitute for missing functional constraints. Liao et al. [8] noted that conventional virtual practice often fails to recalibrate vertical safety margins because of insufficient task-relevant feedback. Recent perspectives on virtual reality-based fall-prevention approaches have similarly emphasized that the absence of physical and contextual constraints can limit the fidelity of motor control and learning outcomes when compared with real-world tasks [31]. In contrast, emerging evidence has highlighted the critical role of augmented information in facilitating motor learning. In particular, the integration of real-time physical feedback into obstacle-avoidance training can significantly enhance safety margins and promote adaptive recalibration of motor output [32]. These observations underscore the importance of providing surrogate signals for physical risk to support simulated training in addressing the gap between motor intention and execution precision.

Ultimately, the potential relevance of simulated training for fall prevention depends on its capacity to elicit authentic biomechanical motor responses. Although the present study focused primarily on foot clearance metrics and did not include a detailed analysis of joint kinematics or kinetics, the results indicate that task repetition in a simulated environment may be insufficient for skill acquisition and should be interpreted with caution if the execution precision deviates substantially from real-world behavior. Our findings suggest that a high-fidelity approximation of actual obstacle-crossing patterns should be prioritized, with particular emphasis on reproducing critical parameters, such as TC_min_ and QCV, through the provision of real-time feedback. The baseline data obtained from healthy young adults in this study provide a necessary reference for the development and calibration of such training protocols. Once biomechanical equivalence is established, these optimized simulated interventions can be extended to older adults and clinical populations to further examine their applicability and limitations as tools for enhancing safety during real-world obstacle crossings.

## 5. Conclusions

This study demonstrated a significant dissociation between motor intention and execution precision during simulated obstacle crossing. Although the simulated environment successfully evoked the overall motor intention to scale gross limb elevation according to the target height, it failed to replicate the precise, fine-grained control required to secure adequate safety margins. This failure was characterized by a systematic underestimation of TC_min_ and elevated motor variability (QCV) patterns, which were most pronounced in the trail limb. These findings suggest that, in the absence of physical risk and its associated sensory feedback, the central nervous system tends to prioritize effort economy over the cognitively demanding fine-tuning of foot trajectories. Consequently, repetitive practice within a risk-free simulated environment alone may be insufficient to reliably promote safe obstacle-crossing strategies. Collectively, these results highlight the importance of biomechanical fidelity and task-relevant feedback as key considerations for the development and evaluation of simulated obstacle-crossing training paradigms.

## Figures and Tables

**Table 1:** Estimated marginal means and 95% confidence intervals for the quartile coefficient of variation of TC parameters in Experiment 2.

Factor		Lead Limb		Trail Limb	
Height	Condition	TC_min_	TC_max_	TC_min_	TC_max_
4%	Actual	0.15 [0.13, 0.17]	0.15 [0.13, 0.17]	0.26 [0.21, 0.32]	0.18 [0.15, 0.21]
	Simulated	0.26 [0.23, 0.30]	0.22 [0.19, 0.25]	0.41 [0.33, 0.50]	0.23 [0.20, 0.27]
8%	Actual	0.14 [0.12, 0.16]	0.14 [0.12, 0.16]	0.34 [0.28, 0.42]	0.22 [0.19, 0.25]
	Simulated	0.23 [0.20, 0.26]	0.21 [0.18, 0.24]	0.50 [0.40, 0.61]	0.27 [0.24, 0.32]
12%	Actual	0.13 [0.12, 0.15]	0.13 [0.12, 0.15]	0.32 [0.26, 0.40]	0.19 [0.16, 0.22]
	Simulated	0.23 [0.21, 0.27]	0.21 [0.19, 0.24]	0.59 [0.48, 0.73]	0.27 [0.24, 0.32]
16%	Actual	0.14 [0.12, 0.15]	0.13 [0.11, 0.15]	0.35 [0.29, 0.43]	0.19 [0.16, 0.22]
	Simulated	0.23 [0.20, 0.26]	0.19 [0.17, 0.22]	0.95 [0.77, 1.17]	0.27 [0.23, 0.31]

Note. *N* = 18. Values were estimated as marginal means averaged across the course conditions. The values in square brackets indicate 95% confidence intervals. TC = toe clearance.

## Data Availability

The data presented in this study are openly available in Mendeley Data at DOI: 10.17632/p3f7bc28cm.1.

## Supplementary Materials

The following supporting information can be downloaded from https://www.mdpi.com/article/10.3390/brainsci16020248/s1: Figure S1: Approach distance during obstacle crossing under actual and simulated conditions; Table S1: Results of linear mixed model analysis for TC of the lead and trail limb in Experiment 1; Table S2: Results of linear mixed model analysis for TC and QCV of the lead limb in Experiment 2; Table S3: Results of linear mixed model analysis for TC and QCV of the trail limb in Experiment 2.

## Abbreviations

The following abbreviations are used in this manuscript:

MTC	Minimum toe clearance
TC	Toe clearance
LMM	Linear mixed model
QCV	Quartile coefficient of variation
AD	Approach distance
