# Supplementary material for: Comparison of Toe Clearance Characteristics Between Simulated Obstacle Crossing Using Visual Height Cues and Actual Obstacle Crossing
Source: Brain Sci. 2026 Feb 23;16(2):248. doi: 10.3390/brainsci16020248 (PMC12938320; doi:10.3390/brainsci16020248)
Supplement: Supplementary file 1 [file brainsci-16-00248-s001.zip › brainsci-4140732-Supplementary Materials.docx]

Article

Comparison of toe clearance characteristics between simulated obstacle crossing using visual height cues and actual obstacle crossing

Mao Kasai ^1^, Yumi Machida ^1^, Miku Washizu ^1^, Kenichi Sugawara ^1^ and Tomotaka Suzuki ^1,^*

^1^ School of Rehabilitation, Kanagawa University of Human Services; suzuki-tm@kuhs.ac.jp

***** Correspondence: suzuki-tm@kuhs.ac.jp; Tel.: +81-46-828-2716

**Supplementary Table S1.** Results of linear mixed model analysis for TC of the lead and trail limb in Experiment 1.

| **Factor** | **Lead limb** | | | | | | **Trail limb** | | | | | |
| --- | --- | --- | --- | --- | --- | --- | --- | --- | --- | --- | --- | --- |
|  | MTC | | | peakTC | | | MTC | | | peakTC | | |
|  | *F* ratio | *p* | η_p_^2^ | *F* ratio | *p* | η_p_^2^ | *F* ratio | *p* | η_p_^2^ | *F* ratio | *p* | η_p_^2^ |
| Covariates |  |  |  |  |  |  |  |  |  |  |  |  |
| Walking time | 49.63 | < 0.001 | 0.027 | 59.10 | < 0.001 | 0.032 | 2.84 | 0.092 | 0.005 | 26.79 | < 0.001 | 0.024 |
| Interactions |  |  |  |  |  |  |  |  |  |  |  |  |
| Height × Course | 1.30 | 0.273 | 0.002 | 0.26 | 0.851 | < 0.001 | 1.11 | 0.342 | 0.001 | 0.62 | 0.600 | 0.001 |
| Main effects |  |  |  |  |  |  |  |  |  |  |  |  |
| Height | 1442.12 | < 0.001 | 0.632 | 1375.13 | < 0.001 | 0.621 | 407.77 | < 0.001 | 0.327 | 913.32 | < 0.001 | 0.521 |
| Course | 56.61 | < 0.001 | 0.022 | 21.22 | < 0.001 | 0.008 | 0.63 | 0.427 | < 0.001 | 4.66 | 0.031 | 0.002 |

*Note*. *N* = 16. Numerator degrees of freedom were 3 for obstacle height and its interactions, and 1 for walking time and course. Denominator degrees of freedom ranged from 611 to 2517 for TC. TC = toe clearance; MTC = minimum toe clearance.

**Supplementary Table S2.** Results of linear mixed model analysis for TC and QCV of the lead limb in Experiment 2.

| **Factor** | **TC_min_** | | | **TC_max_** | | | **QCV (TC_min_)** | | | **QCV (TC_max_)** | | |
| --- | --- | --- | --- | --- | --- | --- | --- | --- | --- | --- | --- | --- |
|  | *F* ratio | *p* | η_p_^2^ | *F* ratio | *p* | η_p_^2^ | *F* ratio | *p* | η_p_^2^ | *F* ratio | *p* | η_p_^2^ |
| Covariates |  |  |  |  |  |  |  |  |  |  |  |  |
| Walking time | 142.46 | < 0.001 | 0.026 | 79.15 | < 0.001 | 0.015 | 1.26 | 0.274 | 0.052 | 3.82 | 0.063 | 0.147 |
| Leg length | 0.84 | 0.373 | 0.050 | 0.31 | 0.585 | 0.019 | 0.48 | 0.499 | 0.032 | 0.27 | 0.614 | 0.017 |
| Interactions |  |  |  |  |  |  |  |  |  |  |  |  |
| Condition × Height × Course | 1.95 | 0.119 | 0.001 | 1.27 | 0.284 | 0.001 | 0.51 | 0.677 | 0.006 | 0.50 | 0.682 | 0.006 |
| Condition × Height | 28.39 | < 0.001 | 0.015 | 20.21 | < 0.001 | 0.010 | 0.11 | 0.956 | 0.001 | 0.11 | 0.952 | 0.001 |
| Condition × Course | 1.25 | 0.263 | < 0.001 | 0.26 | 0.611 | < 0.001 | 0.04 | 0.852 | < 0.001 | 0.44 | 0.506 | 0.002 |
| Height × Course | 2.42 | 0.064 | 0.001 | 3.79 | 0.010 | 0.002 | 0.90 | 0.442 | 0.011 | 0.49 | 0.691 | 0.006 |
| Main effects |  |  |  |  |  |  |  |  |  |  |  |  |
| Condition | 138.49 | < 0.001 | 0.024 | 21.13 | < 0.001 | 0.004 | 170.94 | < 0.001 | 0.388 | 97.59 | < 0.001 | 0.266 |
| Height | 422.14 | < 0.001 | 0.181 | 356.43 | < 0.001 | 0.157 | 1.71 | 0.165 | 0.020 | 2.08 | 0.103 | 0.024 |
| Course | 54.65 | < 0.001 | 0.009 | 31.97 | < 0.001 | 0.006 | 0.99 | 0.320 | 0.004 | 0.20 | 0.652 | 0.001 |

*Note*. *N* = 18. Numerator degrees of freedom were 3 for obstacle height and its interactions, and 1 for all other factors. Denominator degrees of freedom ranged from 5284 to 5742 for TC and from 22 to 270 for QCV. The denominator degrees of freedom for leg length were 16 for TC and 15 for QCV. TC = toe clearance, QCV = quartile coefficient of variation.

**Supplementary Table S3.** Results of linear mixed model analysis for TC and QCV of the trail limb in Experiment 2.

| **Factor** | **TC_min_** | | | **TC_max_** | | | **QCV (TC_min_)** | | | **QCV (TC_max_)** | | |
| --- | --- | --- | --- | --- | --- | --- | --- | --- | --- | --- | --- | --- |
|  | *F* ratio | *p* | η_p_^2^ | *F* ratio | *p* | η_p_^2^ | *F* ratio | *p* | η_p_^2^ | *F* ratio | *p* | η_p_^2^ |
| Covariates |  |  |  |  |  |  |  |  |  |  |  |  |
| Walking time | 219.26 | < 0.001 | 0.050 | 158.57 | < 0.001 | 0.039 | 0.48 | 0.494 | 0.017 | 0.08 | 0.779 | 0.004 |
| Leg length | 0.21 | 0.650 | 0.013 | 0.94 | 0.347 | 0.056 | 0.73 | 0.407 | 0.046 | 0.04 | 0.846 | 0.003 |
| Interactions |  |  |  |  |  |  |  |  |  |  |  |  |
| Condition × Height × Course | 2.42 | 0.064 | 0.001 | 1.75 | 0.154 | 0.001 | 0.69 | 0.558 | 0.008 | 0.30 | 0.829 | 0.003 |
| Condition × Height | 49.80 | < 0.001 | 0.025 | 14.26 | < 0.001 | 0.007 | 5.69 | 0.001 | 0.063 | 0.68 | 0.568 | 0.008 |
| Condition × Course | 11.13 | 0.001 | 0.002 | 3.72 | 0.054 | 0.001 | 1.01 | 0.316 | 0.004 | 2.67 | 0.104 | 0.010 |
| Height × Course | 0.64 | 0.591 | < 0.001 | 3.28 | 0.020 | 0.002 | 0.75 | 0.525 | 0.009 | 0.34 | 0.797 | 0.004 |
| Main effects |  |  |  |  |  |  |  |  |  |  |  |  |
| Condition | 314.19 | < 0.001 | 0.052 | 12.51 | < 0.001 | 0.002 | 97.30 | < 0.001 | 0.267 | 42.82 | < 0.001 | 0.137 |
| Height | 7.56 | < 0.001 | 0.004 | 263.16 | < 0.001 | 0.121 | 16.59 | < 0.001 | 0.164 | 2.86 | 0.037 | 0.033 |
| Course | 3.13 | 0.077 | 0.001 | 53.73 | < 0.001 | 0.009 | 3.03 | 0.083 | 0.012 | 1.90 | 0.170 | 0.007 |

*Note*. *N* = 18. Numerator degrees of freedom were 3 for obstacle height and its interactions, and 1 for all other factors. Denominator degrees of freedom ranged from 3928 to 5734 for TC and from 23 to 270 for QCV. The denominator degrees of freedom for leg length were 16 for TC and 15 for QCV. TC = toe clearance, QCV = quartile coefficient of variation.


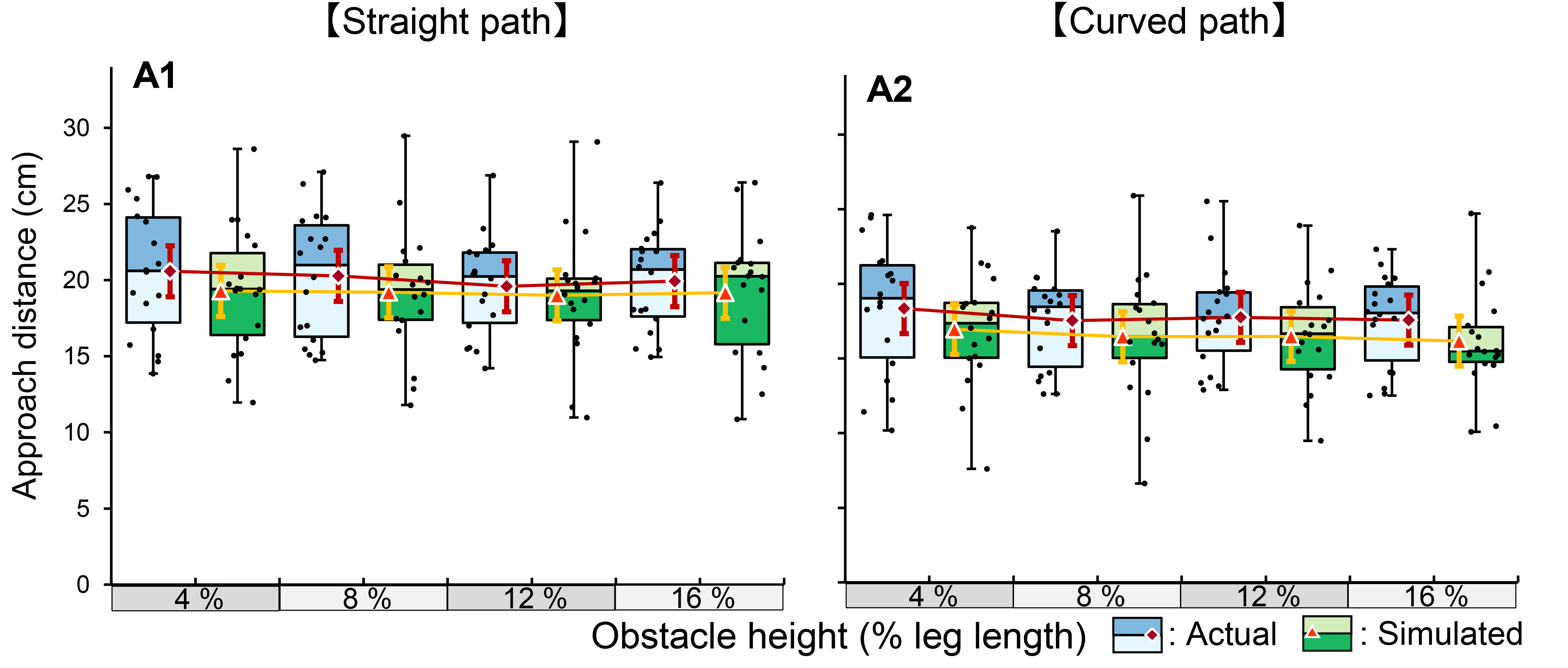


**Figure S1**. Approach distance during obstacle crossing under actual and simulated conditions. Approach distances are shown for the (A1) straight path and (A2) curved path across four obstacle heights (4%, 8%, 12%, and 16% of leg length). Data are presented as jittered boxplots superimposed with estimated marginal means and 95% confidence intervals derived from a linear mixed model.

**Disclaimer/Publisher’s Note:** The statements, opinions and data contained in all publications are solely those of the individual author(s) and contributor(s) and not of MDPI and/or the editor(s). MDPI and/or the editor(s) disclaim responsibility for any injury to people or property resulting from any ideas, methods, instructions or products referred to in the content.
